# Sugar-sweetened beverage prices: Variations by beverage, food store, and neighborhood characteristics, 2017

**DOI:** 10.1016/j.pmedr.2019.100883

**Published:** 2019-04-29

**Authors:** Julien Leider, Lisa M. Powell

**Affiliations:** a Institute for Health Research and Policy, University of Illinois at Chicago, 1747 W. Roosevelt Road, M/C 275, Chicago, IL 60608-1264, USA; b Division of Health Policy and Administration, School of Public Health, University of Illinois at Chicago, 1603 W. Taylor Street, M/C 923, Chicago, IL 60612-4394, USA

**Keywords:** Sugar-sweetened beverages, Price, Sugar-sweetened beverage tax

## Abstract

Sugar-sweetened beverage (SSB) consumption is associated with obesity, type 2 diabetes, and cardiovascular disease. A number of U.S. jurisdictions have levied volume-based specific SSB taxes. This study estimated baseline mean SSB prices across categories and sizes as this will help to determine the percentage increase in price resulting from the imposition of specific taxes.

Data on food store SSB prices were collected in 2017 in Cook County, IL, St. Louis City/County, MO, Oakland, CA, and Sacramento, CA (*N* = 11,767 product-level observations from 581 stores). Data were weighted to represent volume sold by category and size. Mean prices per ounce were computed across categories and sizes. Linear regression models, clustered on store, were run to estimate associations between price per ounce and product characteristics, neighborhood (linked by census tract) characteristics, store type, and site.

Weighted summary statistics show that the mean price of SSBs was 4.8 cents/oz. Soda was least expensive (3.4 cents/oz), followed by sports drinks (4.8 cents/oz), juice drinks (5.2 cents/oz), ready-to-drink tea/coffee (7.8 cents/oz), and energy drinks (19.9 cents/oz). Prices were higher for individual-sized (9.6 cents/oz) compared to family-sized drinks (>1 L/multi-pack; 3.5 cents/oz). Regression results revealed that prices were lower in stores in majority non-Hispanic black tracts and varied by beverage characteristics and store type but not tract-level socioeconomic status.

Given substantial variation in prices by SSB category, a penny-per-ounce SSB tax, if fully passed through, would increase soda prices by 29% versus 5% for energy drinks, highlighting the potential importance of different specific tax rates across beverage categories.

## Highlights

• The mean price of sugar-sweetened beverages (SSBs) was 4.8 cents/oz.
• Prices varied substantially by category and were higher for individual-sized drinks.
• Prices ranged from 3.4 cents/oz for soda to 19.9 cents/oz for energy drinks.
• Prices were lower in majority non-Hispanic black census tracts.
• Prices varied by store type.

## 1 Introduction

Although sugar-sweetened beverage (SSB) consumption has recently declined, in 2013–2014, 50.0% of adults and 60.7% of children drank SSBs on a given day (Bleich et al., 2018). SSBs contributed 6.5% of daily energy intake among adults (Rosinger et al., 2017a) and 7.3% among youth (Rosinger et al., 2017b) in 2011–2014, and SSBs are the leading source of added sugars in the U.S. diet for the population aged 2 years and over (U.S. Department of Health and Human Services and U.S. Department of Agriculture, December, 2015). SSB consumption is higher among non-Hispanic blacks and Hispanics compared to non-Hispanic whites (Rosinger et al., 2017a), and among lower-income compared to higher-income individuals (Ogden et al., 2011), groups which are also more affected by obesity (Ogden et al., 2015; Ogden et al., 2017), diabetes (Beckles and Chou, 2016; Centers for Disease Control and Prevention, 2017), and cardiovascular disease (Fiscella et al., 2009; Graham, 2015). SSB consumption is associated with higher risk of dental caries, obesity, type 2 diabetes, metabolic syndrome, and cardiovascular disease (Bernabé et al., 2014; Hu, 2013; Malik et al., 2010a; Malik et al., 2010b; Vartanian et al., 2007).

The *2015–2020 Dietary Guidelines for Americans* recommend reducing consumption of added sugars, including SSBs (U.S. Department of Health and Human Services and U.S. Department of Agriculture, December, 2015). The Institute of Medicine of the National Academies, the World Health Organization, and public health experts have recommended reducing consumption of SSBs specifically, and have highlighted SSB taxes as a tool to reduce consumption (Brownell and Frieden, 2009; Chaloupka et al., 2011; Institute of Medicine, 2012; World Health Organization, 2015). A number of local jurisdictions in the U.S. have levied taxes on SSBs since Berkeley, California, became the first to do so in 2015 (Center for Science in the Public Interest, 2018). These taxes have all been specific taxes based on volume, ranging from 1 to 2 cents per ounce, rather than ad valorem taxes based on price. It is important to know the baseline mean price of various SSB categories since these may vary substantially and will determine the effective percentage increase in price resulting from the imposition of a specific tax.

Limited evidence is available on SSB prices by beverage category and size, and that which exists documents variation in SSB prices across these characteristics (Powell et al., 2014a). SSB prices may also vary by neighborhood characteristics and store type, given that studies have found evidence that SSB marketing more generally is directed more towards Hispanic, non-Hispanic black, and lower-income youth, as well as Supplemental Nutrition Assistance Program (SNAP) recipients, and the prevalence of SSB price promotions has been found to vary by store type (Harris et al., 2014; Kumar et al., 2015; Moran et al., 2018; Powell et al., 2016; Powell et al., 2014b). In this regard, one study found that soda prices are lower in neighborhoods with a higher concentration of black and Hispanic people and lower socioeconomic status (Kern et al., 2016).

This paper draws on current SSB price data collected in food stores in four U.S. cities/counties in 2017 and estimates multivariable regression models to assess the association of SSB prices with beverage category, size, and sale status, store type, and neighborhood characteristics.

## 2 Methods

### 2.1 Study sample

Data on food store SSB prices were collected in late May and June 2017 in Cook County, IL, St. Louis City and County (hereafter referred to as St. Louis), MO, Oakland, CA, and Sacramento, CA, as part of baseline data collection for a larger study evaluating SSB taxes in Cook County and Oakland. To sample stores for auditing, each site was divided into areas in ArcGIS 10.4. In Cook County and St. Louis, Euclidean allocation based on spatially balanced random seed points was used to divide the sites into 20 areas composed of census tracts (Esri, 2016). In Oakland and Sacramento, which were geographically smaller, census tracts (Oakland) or block groups (Sacramento) were manually allocated to 16 areas, after which a random seed point was chosen for each area for sampling stores.

Stores were audited using our Beverage Tax Food Store Observation Form (Illinois Prevention Research Center, n.d) which was adapted from the previous Bridging the Gap Food Store Observation Form and Illinois Prevention Research Center-Nutrition and Obesity Policy Research Evaluation Network Food Store Observation Form (Rimkus et al., 2013; Singleton et al., 2017). The tool included a range of brands and sizes based on national market shares and common individual and family sizes available for the given products. The mean kappa statistic for the categorical items on our adapted audit tool was 0.84 (almost perfect (Landis and Koch, 1977)), while the mean intraclass correlation coefficient (ICC) was 0.965 for the continuous items. The mean kappa statistic was 0.86 (almost perfect) for availability, 0.80 (substantial) for sale presence, and 0.95 (almost perfect) for sale type. The ICC was 0.997 for regular price, 0.997 for reduced-price sale price, and 0.990 for reduced-price-per-quantity sale price (Li et al., 2018).

Stores were classified into seven types: general merchandise stores, supermarkets, grocery stores, chain and non-chain convenience stores, small discount stores, and drug stores/pharmacies (Powell et al., 2016). General merchandise stores were defined as Walmart, Target, K-Mart, and Meijer (although no Meijer stores were included in the final sample). Supermarkets included stores selling fresh meat with at least two of three staffed service counters: deli, butcher, or bakery. Grocery stores included all other stores selling fresh meat. Limited service stores did not sell fresh meat and included small discount stores (those mentioning discounts or dollar store in their name), drug stores/pharmacies, and convenience stores. Within each area, we aimed to sample stores of every type. Each store was selected based on being the closest store of its type to its area's random seed point by driving distance. After drawing the sample, we reviewed the distributions of sampled stores for each store type across higher- and lower-income areas, based on median household income data from the American Community Survey (U.S. Census Bureau, 2016), and sampled additional stores as needed to ensure adequate distributions across high- and low-income areas. Where a store could not be audited, we replaced it with another store in the same area where possible. Searches were run in Google Maps and Yelp to identify stores and phone calls were conducted where necessary to verify store type. Sample size selection was informed by power analyses revealing that 87 food stores per evaluation site were required to assess tax pass-through for our larger study, in order to detect an 8.5% price increase across beverage categories and sizes. We obtained data for >100 stores in each site.

Audits were conducted at 588 stores across the four sites. The audit form included 63 distinct SSBs, including different sizes of the same brands. This allowed for the potential observation of 37,044 prices. Prices were collected from shelf tags, although for certain products data collectors were instructed to ask for the price if it was not shown. Availability was determined for 13,815 product-level observations (22,496 observations were not available/sold in stores and data on availability were missing for 733 observations). Another 138 observations had to be excluded because of missing information on whether the product was on sale, and the price was missing for another 1910 observations, leaving 11,767 product-level observations from 581 stores in the final analytical sample. This study was approved by the Institutional Review Board of the University of Illinois at Chicago.

### 2.2 Measures

The price measure was computed to reflect each product's shelf price and equaled the product's sale price if it was on sale and its regular price if it was not on sale. For this purpose, only reduced-price and reduced-price-per-quantity sales were considered, as other sale types (such as “buy one, get one” sales) do not have a constant price per unit. SSBs were classified into five categories: non-100% juice drinks (hereafter referred to as juice drinks), soda, sports drinks, energy drinks, and ready-to-drink tea and coffee. The beverages were classified into two sizes, individual and family sized, where family sized was defined as >1 L for a single item or a multi-pack of any size.

Data on prices were linked to neighborhood characteristics from the American Community Survey based on census tract (U.S. Census Bureau, 2016). Characteristics included median household income, the percent of the population below 125% of the poverty level, and majority race/ethnicity, which was coded as ≥50% non-Hispanic white, ≥50% non-Hispanic black, ≥50% Hispanic, and “other” (tracts that were not ≥50% non-Hispanic white, non-Hispanic black, or Hispanic).

### 2.3 Statistical analysis

Our audit form was designed to capture a range of brands and sizes and thereby overrepresented certain beverage categories and sizes relative to their proportion of volume sold (e.g., energy drinks, which represent a relatively small proportion). To address this, descriptive statistics and regressions were weighted based on the overall distribution of volume sold by beverage category and size. Specifically, Nielsen retail scanner data were used to compute the distribution of SSB volume sold by beverage category and size across the four sites (Cook County, St. Louis, Oakland, and Sacramento and a two-mile buffer surrounding each site) between June 2016 and May 2017. This year-long time period was chosen to pre-date Cook County and Oakland tax implementation while accounting for potential seasonal variation in the volume distribution. Sizes larger than those represented on the audit form for a given beverage category were excluded for purposes of computing the volume distribution.

Weighted mean prices per ounce were computed by beverage category and size. Multivariable linear regression models were run for SSBs overall and each SSB category linking price per ounce to beverage category, size, and sale status, store type, and neighborhood characteristics, controlling for site. Models were weighted to the overall distribution of volume sold by beverage category and size and clustered on store identifier with robust standard errors. Variance inflation factors (VIFs) were computed for all independent variables in all models to check for collinearity; all VIFs were below 4 and mean VIFs for all models were below 2, well below standard rule of thumb thresholds for multicollinearity (StataCorp, 2013). Adjusted means were computed from these models showing mean predicted prices per ounce for each store type and overall. Data entry was undertaken using a Research Electronic Data Capture (REDCap) database (UIC Center for Clinical Translational Science, 2018). Data cleaning and analyses were conducted in Stata/SE 13.1.

## 3 Results

Table 1 shows descriptive statistics for the analytical sample. The sample was distributed across the four sites and all seven store types. The sampled stores were located in tracts with a range of racial/ethnic makeups, with a plurality of stores (246 out of 581) located in majority non-Hispanic white census tracts. The mean tract-level median household income was $57,534 (range of $11,295–$169,048) for the sampled stores, with a mean of 24.0% (range of 0%–83.8%) of the population below 125% of the poverty level. We have a substantial number of price observations across SSB categories and sizes. The largest number of observations are for soda (5220), followed by energy drinks (2276), sports drinks (1931), ready-to-drink tea and coffee (1454), and juice drinks (886). Weighted product-level observations show that soda represented 54.0% of volume sold across the four sites, followed by juice drinks at 23.3%, while energy drinks represented just 2.8%. Although we audited similar numbers of individual- (6504) and family-sized (5263) products, weighted family-sized SSB observations represented 78.3% of volume. Products were on sale in 4401 observations, corresponding to a weighted percentage of nearly 40%.

**Table 1:** Characteristics of the analytical sample of food store sugar-sweetened beverage price observations in Cook County, IL, St. Louis City and County, MO, Oakland, CA, and Sacramento, CA, in 2017.

Characteristics	% or mean (SD)
**Store-level (*****N***** = 581 stores)**	
Site	
Cook County, IL (*n* = 167)	28.7
St. Louis City and County, MO (*n* = 165)	28.4
Oakland, CA (*n* = 125)	21.5
Sacramento, CA (*n* = 124)	21.3
Store type	
General merchandise (*n* = 36)	6.2
Supermarket (*n* = 116)	20.0
Grocery store (*n* = 66)	11.4
Chain convenience store (*n* = 133)	22.9
Non-chain convenience store (*n* = 119)	20.5
Small discount store (*n* = 33)	5.7
Drug store/pharmacy (*n* = 78)	13.4
Majority race/ethnicity	
Majority (≥50%) non-Hispanic white (*n* = 246)	42.3
Majority (≥50%) non-Hispanic black (*n* = 106)	18.2
Majority (≥50%) Hispanic (*n* = 55)	9.5
Other (*n* = 174)	29.9
Median household income	57,534.4 (30,564.6)
% of the population below 125% of the poverty level	24.0 (15.6)
**Product-level (*N* = 11,767 products)**	
Beverage category	
Soda (*n* = 5220)	54.0
Sports drink (*n* = 1931)	11.7
Energy drink (*n* = 2276)	2.8
Ready-to-drink tea and coffee (*n* = 1454)	8.2
Juice drink (non-100% juice) (*n* = 886)	23.3
Size	
Individual (*n* = 6504)	21.7
Family (>1 L or multi-pack) (*n* = 5263)	78.3
On sale (*n* = 4401)	38.8

Product-level percentages are weighted to be representative of the volume sold by beverage category and size in all four sites, and the two-mile buffers surrounding them, in June 2016–May 2017. Unweighted *n*'s shown in parentheses.

Table 2 shows the weighted mean price per ounce of SSBs overall and across categories and sizes. The mean price of SSBs was 4.8 cents/oz. Prices were higher for individual- than family-sized drinks, both overall (9.6 cents/oz for individual-sized SSBs versus 3.5 cents/oz for family-sized SSBs) and across all categories of SSBs. Soda was the least expensive of the SSB categories, at 3.4 cents/oz; and, it was the lowest cost SSB of any form at only 2.9 cents/oz for the family size. Sports drinks were the least expensive individual-sized SSB at 5.6 cents/oz. Overall, sports drinks (4.8 cents/oz), juice drinks (5.2 cents/oz), and ready-to-drink tea and coffee (7.8 cents/oz) were more expensive than soda. Energy drinks were by far the most expensive SSB at 19.9 cents/oz. To provide additional context, Table 2 also shows mean prices for artificially-sweetened beverages (ASBs) and unsweetened beverages. Mean ASB prices (4.5 cents/oz overall) were similar to corresponding SSB prices. Unsweetened beverages were cheaper (2.8 cents/oz), particularly bottled water (1.6 cents/oz). However, 100% juice (7.9 cents/oz) was more expensive than SSB juice drinks.

**Table 2:** Mean price in cents per ounce of sugar-sweetened and other beverages in food stores in Cook County, IL, St. Louis City and County, MO, Oakland, CA, and Sacramento, CA, in 2017.

Categories	Overall	Individual size	Family size
SSBs (*n* = 11,767; 6504; 5263)	4.8 (4.2)	9.6 (5.9)	3.5 (2.2)
Soda (*n* = 5220; 2013; 3207)	3.4 (1.9)	7.8 (1.6)	2.9 (1.2)
Sports drink (*n* = 1931; 1486; 445)	4.8 (2.2)	5.6 (2.3)	3.4 (0.9)
Energy drink (*n* = 2276; 1788; 488)	19.9 (6.3)	20.2 (6.3)	17.2 (5.9)
Ready-to-drink tea and coffee (*n* = 1454; 996; 458)	7.8 (6.3)	10.2 (6.7)	6.3 (5.5)
Juice drink (non-100% juice) (*n* = 886; 221; 665)	5.2 (3.3)	12.3 (2.3)	4.0 (1.4)
ASBs (*n* = 6560; 3423; 3137)	4.5 (4.1)	9.4 (5.9)	3.2 (2.0)
Soda (*n* = 3916; 1463; 2453)	3.7 (2.2)	8.1 (1.4)	3.0 (1.2)
Sports drink (*n* = 623; 455; 168)	5.1 (2.2)	5.4 (2.3)	3.7 (1.1)
Energy drink (*n* = 1795; 1436; 359)	19.6 (6.2)	20.2 (6.1)	17.0 (5.9)
Unsweetened (*n* = 5213; 1992; 3221)	2.8 (2.5)	9.2 (3.5)	2.4 (1.6)
Water (*n* = 1150; 1048; 102)	1.6 (1.6)	7.0 (1.9)	1.2 (0.3)
Milk (*n* = 2907; 284; 2623)	3.5 (1.9)	12.5 (2.7)	3.3 (1.0)
100% Juice (*n* = 776; 378; 398)	7.9 (3.3)	13.3 (2.2)	6.4 (1.6)

Mean prices per ounce are shown with standard deviations in parentheses, weighted to be representative of volume sold by beverage sweetener status (SSB/ASB/unsweetened), category, and size in all four sites, and the two-mile buffers surrounding them, in June 2016–May 2017. Overall, individual size, and family size sample sizes for each row shown in parentheses separated by semicolons.

SSB, sugar-sweetened beverage; ASB, artificially-sweetened beverage.

Table 3 shows results from multivariable linear regression models of the association of SSB price per ounce with beverage category, size, and sale status, store type, and neighborhood characteristics. In the model for all SSBs, the beverage categories show a similar pattern to the unadjusted means in Table 2, except that after adjusting for these characteristics sports drinks were slightly less expensive than soda. All pairwise differences among beverage categories from this model were significant at *p* < .001. As in Table 2, family-sized beverages were consistently less expensive than individual-sized beverages (overall, −4.45 cents/oz, 95% CI = −4.60, −4.31). This quantity discount held across all categories of SSBs with the largest quantity discounts for soda and juice drinks. SSBs that were on sale were almost one cent per ounce cheaper (−0.89 cents/oz, 95% CI = −1.00, −0.77), although this varied across categories, with the largest depth of sale in absolute terms for energy drinks (−3.74 cents/oz, 95% CI = −4.31, −3.17) and the largest in relative terms (based on adjusted mean price per ounce from the model) for sports drinks (−35%), and no significant association between sale status and price per ounce for ready-to-drink tea and coffee.

**Table 3:** Association between sugar-sweetened beverage price in cents per ounce and product, store, and neighborhood characteristics in food stores in Cook County, IL, St. Louis City and County, MO, Oakland, CA, and Sacramento, CA, in 2017.

	All SSBs (*N* = 11,767)	Soda (*n* = 5220)	Sports drinks (*n* = 1931)	Energy drinks (*n* = 2276)	Ready-to-drink tea and coffee (*n* = 1454)	Juice drinks (Non-100% juice) (*n* = 886)
	Coef. (95% CI)	Coef. (95% CI)	Coef. (95% CI)	Coef. (95% CI)	Coef. (95% CI)	Coef. (95% CI)
Beverage category (Ref: Soda)						
Sports drink	−0.84*** (−0.97, −0.72)					
Energy drink	12.75*** (12.54, 12.97)					
Ready-to-drink tea and coffee	3.10*** (2.89, 3.31)					
Juice drink (non-100% juice)	1.69*** (1.55, 1.83)					
Size (Ref: Individual)						
Family (>1 L or multi-pack)	−4.45*** (−4.60, −4.31)	−4.75*** (−4.85, −4.65)	−1.05*** (−1.23, −0.88)	−2.86*** (−3.20, −2.52)	−3.98*** (−4.44, −3.51)	−6.95*** (−7.41, −6.50)
Sale status						
On sale	−0.89*** (−1.00, −0.77)	−0.59*** (−0.68, −0.49)	−1.69*** (−1.88, −1.50)	−3.74*** (−4.31, −3.17)	0.25 (−0.50, 0.99)	−1.07*** (−1.33, −0.81)
Store type (Ref: Supermarket)						
General merchandise	−0.52*** (−0.70, −0.35)	−0.24** (−0.40, −0.08)	−0.71*** (−0.94, −0.49)	−2.05*** (−2.65, −1.45)	0.58 (−0.01, 1.18)	−1.11*** (−1.39, −0.82)
Grocery store	−0.68*** (−0.95, −0.40)	−0.44*** (−0.60, −0.28)	−0.87*** (−1.26, −0.49)	−2.00*** (−3.18, −0.82)	−0.64 (−1.92, 0.64)	−0.74* (−1.32, −0.15)
Chain convenience store	0.62*** (0.48, 0.77)	0.18** (0.07, 0.30)	1.92*** (1.72, 2.13)	1.23*** (0.84, 1.61)	0.47 (−0.20, 1.15)	1.36*** (0.71, 2.01)
Non-chain convenience store	0.08 (−0.15, 0.30)	−0.41*** (−0.58, −0.23)	1.22*** (0.91, 1.54)	0.89** (0.22, 1.56)	−0.56 (−1.66, 0.54)	−0.02 (−0.82, 0.78)
Small discount store	−0.79*** (−0.93, −0.65)	−0.42*** (−0.53, −0.31)	−1.12*** (−1.47, −0.77)	−1.25 (−2.91, 0.41)	−1.38*** (−2.12, −0.63)	−0.74*** (−1.04, −0.44)
Drug store/pharmacy	0.37*** (0.24, 0.49)	0.21*** (0.12, 0.30)	0.86*** (0.53, 1.18)	0.24 (−0.20, 0.67)	−0.01 (−0.53, 0.51)	1.11*** (0.86, 1.36)
Race/ethnicity (Ref: Majority (≥50%) NH white)						
Majority (≥50%) NH black	−0.27** (−0.45, −0.08)	−0.17* (−0.30, −0.03)	−0.27 (−0.58, 0.03)	−0.87* (−1.61, −0.13)	−0.53 (−1.30, 0.24)	−0.25 (−0.60, 0.09)
Majority (≥50%) Hispanic	−0.09 (−0.33, 0.15)	−0.12 (−0.28, 0.04)	−0.16 (−0.54, 0.23)	−0.75 (−1.73, 0.23)	−0.15 (−1.31, 1.01)	0.07 (−0.46, 0.60)
Other	−0.06 (−0.20, 0.08)	−0.05 (−0.16, 0.06)	−0.23* (−0.45, −0.01)	−0.54* (−0.97, −0.11)	0.21 (−0.32, 0.75)	−0.03 (−0.29, 0.23)
Median HH income (units of $10 k)	0.02 (−0.01, 0.04)	0.02* (0.00, 0.04)	0.00 (−0.03, 0.03)	0.02 (−0.05, 0.10)	0.02 (−0.09, 0.12)	0.02 (−0.03, 0.07)
% population below 125% of the poverty level	0.00 (−0.01, 0.01)	0.00 (−0.01, 0.00)	0.00 (−0.00, 0.01)	0.02 (−0.00, 0.04)	−0.02 (−0.05, 0.00)	0.00 (−0.01, 0.02)
Site (Ref: Cook County, IL)						
St. Louis City and County, MO	0.00 (−0.12, 0.11)	0.09 (−0.00, 0.18)	−0.09 (−0.27, 0.08)	0.07 (−0.40, 0.53)	−0.11 (−0.56, 0.34)	−0.12 (−0.35, 0.11)
Oakland, CA	0.65*** (0.41, 0.88)	0.39*** (0.22, 0.55)	0.44** (0.15, 0.72)	0.66 (−0.01, 1.34)	0.69 (−0.20, 1.57)	1.00*** (0.55, 1.46)
Sacramento, CA	0.30*** (0.13, 0.47)	0.35*** (0.23, 0.46)	0.07 (−0.17, 0.31)	−0.06 (−0.57, 0.45)	0.24 (−0.41, 0.89)	0.49* (0.10, 0.88)
Constant	7.59*** (7.27, 7.91)	7.78*** (7.54, 8.01)	5.85*** (5.43, 6.27)	21.24*** (20.34, 22.14)	10.41*** (9.17, 11.65)	11.22*** (10.53, 11.91)

Regressions are at the product level and are weighted to be representative of volume sold by beverage category and size in all four sites, and the two-mile buffers surrounding them, in June 2016–May 2017. Models are clustered on store identifier with robust standard errors.

SSB, sugar-sweetened beverage; NH, non-Hispanic.

Bold denotes statistical significance (* *p* < .05, ** *p* < .01, *** *p* < .001).

Turning to neighborhood characteristics, prices in majority non-Hispanic black census tracts were lower for SSBs overall (−0.27 cents/oz, 95% CI = −0.45, −0.08, 5.6% of the adjusted mean price of SSBs) and for soda (−0.17 cents/oz, 95% CI = −0.30, −0.03, 5.0% of the adjusted mean) and energy drinks (−0.87 cents/oz, 95% CI = −1.61, −0.13, 4.4% of the adjusted mean) specifically compared to prices in majority non-Hispanic white census tracts. There were no significant associations between SSB prices and median household income or poverty, except a small positive association between income and the price of soda. While there were no significant differences in prices between the two Midwestern sites, prices were higher in the two cities in California compared to Cook County, suggesting regional variation in prices.

Table 3 also shows that SSB prices differed by store type. Compared to prices in supermarkets, SSB prices in general merchandise stores, grocery stores and small discount stores were lower by about a half to three quarters of a cent per ounce (−0.52 to −0.79); whereas SSB prices were, on average, higher by a third and two thirds of a cent at drug stores/pharmacies and chain convenience stores, respectively. Whereas there was no significant difference in prices between non-chain convenience stores and supermarkets, the pattern differed by beverage category; soda was less expensive in non-chain convenience stores but there was a price premium in such stores for sports drinks and energy drinks. Ready-to-drink tea and coffee exhibited the least variation in price by store type, with the exception that it was significantly cheaper at small discount stores compared to supermarkets.

Table 4 presents adjusted mean prices per ounce for SSBs by store type, based on the models in Table 3. Prices for SSBs overall were highest at chain convenience stores (5.5 cents/oz, 95% CI = 5.3, 5.6), and chain convenience stores had at or near the highest prices for specific categories of SSB as well. Supermarkets, non-chain convenience stores, and drug stores/pharmacies had the next highest prices. Prices were notably lower at general merchandise and grocery stores, and were lowest for small discount stores (4.1 cents/oz, 95% CI = 3.9, 4.2).

**Table 4:** Adjusted mean sugar-sweetened beverage price in cents per ounce by store type in food stores in Cook County, IL, St. Louis City and County, MO, Oakland, CA, and Sacramento, CA, in 2017.

	All SSBs (*N* = 11,767)	Soda (*n* = 5220)	Sports drinks (*n* = 1931)	Energy drinks (*n* = 2276)	Ready-to-drink tea and coffee (*n* = 1454)	Juice drinks (Non-100% Juice) (*n* = 886)
	Adj. Mean (95% CI)	Adj. Mean (95% CI)	Adj. Mean (95% CI)	Adj. Mean (95% CI)	Adj. Mean (95% CI)	Adj. Mean (95% CI)
General merchandise	4.3 (4.2, 4.5)	3.2 (3.1, 3.4)	3.9 (3.7, 4.1)	17.7 (17.2, 18.3)	8.4 (8.0, 8.8)	4.2 (3.9, 4.4)
Supermarket	4.9 (4.7, 5.0)	3.4 (3.4, 3.5)	4.6 (4.5, 4.8)	19.8 (19.5, 20.1)	7.8 (7.4, 8.2)	5.3 (5.0, 5.6)
Grocery store	4.2 (3.9, 4.4)	3.0 (2.9, 3.1)	3.7 (3.4, 4.1)	17.8 (16.7, 18.9)	7.2 (6.0, 8.3)	4.6 (4.0, 5.1)
Chain convenience store	5.5 (5.3, 5.6)	3.6 (3.5, 3.7)	6.5 (6.4, 6.7)	21.0 (20.7, 21.3)	8.3 (7.7, 8.9)	6.7 (6.0, 7.3)
Non-chain convenience store	4.9 (4.7, 5.1)	3.0 (2.9, 3.2)	5.8 (5.5, 6.1)	20.7 (20.0, 21.3)	7.3 (6.2, 8.3)	5.3 (4.5, 6.0)
Small discount store	4.1 (3.9, 4.2)	3.0 (2.9, 3.1)	3.5 (3.2, 3.8)	18.5 (16.9, 20.2)	6.4 (5.9, 7.0)	4.6 (4.3, 4.8)
Drug store/pharmacy	5.2 (5.1, 5.3)	3.7 (3.6, 3.7)	5.5 (5.1, 5.8)	20.0 (19.6, 20.4)	7.8 (7.4, 8.2)	6.4 (6.2, 6.6)
Overall	4.8 (4.7, 4.9)	3.4 (3.3, 3.5)	4.8 (4.7, 5.0)	19.9 (19.7, 20.1)	7.8 (7.6, 8.0)	5.2 (5.0, 5.5)

Adjusted means are shown from regression models at the product level weighted to be representative of volume sold by beverage category and size in all four sites, and the two-mile buffers surrounding them, in June 2016–May 2017. Models controlled for beverage category, size, sale status, tract-level race/ethnicity, income, and poverty, and site, in addition to store type, and were clustered on store identifier with robust standard errors.

SSB, sugar-sweetened beverage.

## 4 Discussion

This paper contributes to a limited existing literature by providing current data on SSB prices, including by SSB category and store type. A previous study that estimated SSB prices using 2010–2012 data found somewhat higher prices (5.9 cents/oz versus 4.8 cents/oz) (Powell et al., 2014a). Despite some differences between our study and the previous study in terms of geographic coverage (national versus four U.S. sites), outlet types (stores and fast food restaurants versus stores alone), and products covered (narrower versus larger range), we found similar patterns of relative prices across beverage categories. Another more recent study collected store audit data on prices for an evaluation of Berkeley, CA's SSB tax (Falbe et al., 2015). That study's estimates of pretax, 2014 prices for individual-sized SSBs (on average 10.35–10.91 cents/oz) were roughly similar to our finding of 9.6 cents/oz for individual-sized SSBs, despite the earlier study being based on a more limited set of products. The study collected limited data on prices for larger sizes but did not report price estimates for those sizes.

Unlike ad valorem taxes, specific taxes based on volume are not affected by quantity discounts and cannot be avoided by switching to cheaper brands (Chriqui et al., 2013). Since SSB prices vary significantly across categories, specific taxes have different effective tax rates across these beverages. This is important given the implications for differential effects on consumption by category. For example, based on our study findings, a penny-per-ounce tax, if fully passed through, would raise the price of soda by 29%, sports drinks by 21%, juice drinks by 19%, and ready-to-drink tea and coffee by 13%, but would only raise the price of energy drinks by 5%. However, past estimates of pass-through have varied, and this will affect the percentage increase in price associated with a specific tax. For instance, a study of Berkeley, CA's SSB tax found 47% pass-through for individual-sized SSBs, while a study of Philadelphia, PA's tax found almost full pass-through at 93% (Cawley et al., 2018; Falbe et al., 2015). Furthermore, some studies have found differences in pass-through by SSB category, which will affect differences in the percentage increase in price associated with a specific tax. For instance, a study in Berkeley found 69% pass-through for soda but 47% pass-through for fruit-flavored beverages, while a study in Mexico found more than full pass-through for carbonated SSBs but less than full pass-through for non‑carbonated SSBs (Colchero et al., 2015; Falbe et al., 2015). While soda is the most consumed SSB, consumption of sports/energy drinks and sweetened coffee/tea has risen as consumption of soda has declined. This further highlights the potential importance of differences in price increases by category (Bleich et al., 2018; Han and Powell, 2013; Kit et al., 2013).

This study also finds significantly lower prices for family-sized as opposed to individual-sized SSBs, across all categories of SSB. This suggests that volume-based taxes will have the largest impact on family-sized drinks, both due to the greater volume involved and because the tax represents a larger percentage of the price. This could encourage consumers to switch to smaller-sized packages of SSBs, which could lead to lower SSB consumption given past studies showing that smaller portion sizes are associated with reduced food consumption (Ledikwe et al., 2005; Roberto and Pomeranz, 2015; Schwartz et al., 2012). However, this could be attenuated if pass-through were higher for individual-sized SSBs, as has been found, for example, in some studies in Mexico and Berkeley, CA (Colchero et al., 2015; Falbe et al., 2015).

We found significant variations in SSB prices by store type with lower prices for SSBs in general merchandise stores, grocery stores and small discount stores compared to supermarkets and relatively higher prices in chain convenience stores and drug stores/pharmacies. Non-chain convenience stores had relatively lower prices for soda but higher prices for sports and energy drinks. Such differences will have implications for different populations based on both the availability of different types of stores and their shopping patterns.

While this study found that, controlling for store types, there were almost no associations between income or poverty and SSB prices, significant associations were found with neighborhood racial/ethnic composition. In particular, prices of SSBs were lower overall and for soda and energy drinks specifically in majority non-Hispanic black census tracts compared to majority non-Hispanic white tracts. This is consistent with other studies showing different forms of SSB marketing are directed more towards non-Hispanic black youth and one study showing soda prices are lower in neighborhoods with a higher concentration of black and Hispanic people (Harris et al., 2014; Kern et al., 2016; Kumar et al., 2015; Powell et al., 2014b). Such pricing patterns are of concern given the greater burden of obesity, diabetes, and cardiovascular disease among non-Hispanic blacks, and the association of SSB consumption with these conditions (Centers for Disease Control and Prevention, 2017; Graham, 2015; Hu, 2013; Malik et al., 2010a; Malik et al., 2010b; Ogden et al., 2015; Vartanian et al., 2007).

### 4.1 Study limitations and strengths

This study provides important updated data on SSB prices; however, it is subject to several limitations. First, data were only collected in four sites that are part of a larger tax evaluation project. And, we found variation in SSB prices across regions, so future studies should explore this further and collect data from a broader set of locations. Second, while we collected prices for a wide range of products, our form was still necessarily limited and represented the major brands of SSBs. Third, while we collected data from a range of store types and found significant variation in prices by store type, we lacked information on the distribution of stores or sales by store type and were unable to weight our estimates accordingly or assess the representativeness of our sample. Fourth, because data were collected shortly before taxes went into effect in two of the four sites (Cook County and Oakland), it is possible retailers had already changed their prices in advance of tax implementation. Strengths of this study include the large sample size in terms of both stores and product observations and the range of SSB categories and sizes for which data were collected.

## 5 Conclusion

This study estimated that SSB prices are, on average, 4.8 cents/oz and that a specific penny-per-ounce excise tax if fully passed through to prices would raise the price of SSBs by about 21%. The substantial range in SSB prices by category (from a low of 3.4 cents/oz for soda to 19.9 cents/oz for energy drinks) and the associated differential impact of a penny-per-ounce tax (increasing soda prices by 29% versus 5% for energy drinks) highlights the potential importance of considering different specific tax rates based on SSB categories. The study results also demonstrate that owing to quantity discounts, specific taxes will have greater impacts on family-size SSBs, which may help to further reduce consumption.
